# Exploring predictors of psychological preparedness for flood victims: A conceptual framework for Malaysia

**DOI:** 10.12688/f1000research.160231.1

**Published:** 2025-02-12

**Authors:** Nur Farhana Lyana Ameruddin, Elizaveta Berezina, Chin Choo Yap

**Affiliations:** 1 Department of Psychology, School of Medical and Life Sciences, Sunway University, Bandar Sunway, Selangor, 47500, Malaysia

**Keywords:** psychological preparedness, flood, Malaysia, natural disaster

## Abstract

**Background:**

Flooding is one of the most frequent and damaging disasters in Malaysia with major social, economic, and psychological consequences. Compared to material and logistical preparedness, the psychological aspects of disaster management are not given much emphasis.

**Methods:**

This conceptual paper employs a systematic literature review to understand the factors that predict Malaysians psychological preparedness for floods. The literature search was conducted using databases such as PubMed, Academic Search Ultimate, Directory of Open Access Journals, Supplemental Index and Google Scholar with keywords including “disaster preparedness, flood, natural disaster and psychological preparedness”. Articles were included if they focused on preparedness towards disasters as well as examined coping mechanisms, perceptions and preparedness behaviours in the context of flood-related crises and excluded if they lack methodological rigor. The review synthesized findings using a thematic analysis approach, identifying psychological resilience and coping strategies in flood-affected populations, the role of social support networks in preparedness and recovery as well as mental health implications of floods, including anxiety, trauma, and post-disaster recovery. This synthesis informed the development of a conceptual model to address factors that predict Malaysians psychological preparedness for floods.

According to contemporary theories of catastrophe risk reduction and psychological resilience, critical variables included are risk perception, outcome expectancy, self-efficacy, anxiety, and social support.

**Results:**

It was proposed that these factors work together to determine an individual’s psychological preparedness, enhancing their ability to cope with the psychological and emotional strain of a flood disaster.

**Conclusions:**

By offering these perspectives, the study contributes to the limited academic discussion on psychological preparedness in Malaysia, which aims to improve the psychological resilience of communities vulnerable to flooding. The proposed framework emphasizes the importance of incorporating psychological preparedness into existing disaster management strategies to reduce the overall damage caused by floods.

## Introduction

Many countries, such as the United States, Australia, and the Philippines, are experiencing severe disruption and upheaval due to the increasing frequency and intensity of natural disasters such as United States, Australia and the Philippines ( Bozick, 2021; Cowlishaw et al., 2024; Rocha et al., 2021; Shah et al., 2017). Among the most common and destructive disasters is flooding, which seriously affects both the economy and human life ( Maurya et al., 2021). Owing to the significant effects of climate change, researchers predict that flooding will become more likely in many parts of the world, including Malaysia ( Shah et al., 2017; Singh & Pandey, 2021).

Floods in Malaysia can be broadly categorized into flash floods and monsoon floods. Flash floods occur rapidly and unexpectedly during everyday events, which makes them particularly vulnerable to the population, especially travellers ( Ruin et al., 2009). In contrast, monsoonal floods are seasonal. Southwest monsoon winds from May to September, and the Northeast monsoon winds from November to March are more predictable and seasonal ( Buslima et al., 2018). However, the most frequent and damaging hydro-meteorological events in Malaysian cities, especially Kuala Lumpur and Kajang, are flash floods ( Bari et al., 2021).

The impact of floods on Malaysia has been phenomenal. In 2024, more than 122,000 people were forced out of their homes because of the massive flood caused by continuous rain in Malaysia’s northern states ( CNA, 2024). The floods that struck Malaysia in December 2006 and January 2007 were thought to be the worst in history. Over 90,000 people were evacuated from Johor state due to severe flooding, while over 20,000 people from the districts of Pekan and Rompin in another state were also relocated to safer areas. Owing to prolonged periods of excessive rainfall and changes in land management, floods also unexpectedly struck the eastern regions of Malaysia in 2013. According to Abdul Rahman (2014), this factor has caused an increase in the frequency, size, and dimensions of floods in certain locations.

The December 2021 floods across six states displaced 19,711 families, with the Klang Valley experiencing the greatest economic impact ( Aiman, 2021; Mahalingam, 2021). The floods in Shah Alam from December 18 to 22 significantly affected Q4 earnings, highlighting the severe consequences of flooding, including 100,000 evacuations, loss of lives, and financial fallout, underscoring their profound social and economic impact. Malaysia is susceptible to flooding owing to environmental exposure ( Rehan & Zakaria, 2021). Posttraumatic Stress disorder (PTSD), sadness, anxiety, and even suicide are among the psychological effects of natural catastrophes on human mental well-being ( Amiri & Jahanitabesh, 2022). This highlights the need to carefully consider psychological preparedness for natural disasters, particularly floods that occur in Malaysia.

### Problem statement

Disaster preparedness in Malaysia aims to raise awareness, support response, and rescue operations. Key agencies include the Department of Irrigation and Drainage (DID), the Malaysian Meteorological Department (MMD), and the Malaysian Remote Sensing Agency (MRSA). DID handles early warning systems, MMD provides weather reports, and MRSA offers advanced remote-sensing technology. Short Message Service (SMS) notifications are suggested for the better dissemination of disaster information. Real-time rainfall and river water level information can be accessed via the Infobanjir website. Following the December 2014 flood tragedy, the Malaysian government implemented proactive measures including an early warning system and funding for flood mitigation projects ( Noorhashirin et al., 2016). A game-based approach was developed to improve flood preparedness among youth by incorporating e-books, board games, and question cards ( Ridzuan et al., 2023). The Metaverse environment includes interactive quizzes, escape games, and real-time access to flood management websites ( Sa Don et al., 2023). These measures aim to reduce flood damage, improve emergency response, reduce death rates, and mitigate stress associated with natural disasters.

The government has established physical preparedness in terms of infrastructure, early warning systems, and flood mitigation efforts; however, psychological preparedness is just as important. Reser (1996) raised interest in psychological preparedness as a risk management tool for natural disasters by arguing that individual preparedness was important for reducing traumatic stress during natural disasters, in addition to common emotions felt during disasters, such as fear, lack of control, and concern. To maintain an appropriate cognitive function and conduct, psychological preparation serves the purpose of controlling reactions to a scenario ( Morrissey & Reser, 2003; Randrianarisoa et al., 2021; Reser, 1996). A good level of psychological preparedness also acts as a protective layer against distress during disasters ( Said & Chiang, 2020).

### Research questions

The current study will be exploring the following research questions:

1) What are the key predictors of psychological preparedness towards flood among Malaysian population?
2) How do individual and community-level factors influence psychological preparedness for floods?

### Importance of psychological preparedness

As psychological preparedness is an integral part of disaster preparedness, it is crucial because it not only affects how one responds physically in various circumstances that require action, but also plays a major role in reducing the physical, social, psychological, and sociocultural harm that comes with disasters, helping people adjust and feel safer ( Blake et al., 2017; Margaritova, 2008). When a tragedy strikes, psychological preparedness enables people to act appropriately and quickly ( Reser & Morrissey, 2009; Suhaimi & Marzuki, 2016). Thus, the sum of internal awareness, readiness, and anticipation is intricately formed by personality factors such as anxiety, self-efficacy, optimism, and self-esteem ( Malkina-Pykh & Pykh, 2013). Ignoring psychological aspects heightens stress, anxiety, and depression levels among flood victims. The World Health Organization (WHO) highlights that the psychological toll of floods often leaves a trail of mental health issues, which can persist long after physical damage is repaired ( WHO, 2023). Without psychological preparedness, individuals may panic or make poor decisions during flood events. This can increase the risk of injury and death. Studies have shown that psychological preparedness helps individuals to remain calm and make better decisions under pressure ( Suhaimi & Marzuki, 2016). Psychological preparedness fosters a sense of community and mutual support. When this aspect is neglected, social cohesion can weaken, making it harder for communities to effectively work together during and after a disaster ( Suhaimi & Marzuki, 2016). Without psychological preparedness, individuals and authorities may experience heightened stress and anxiety, leading to delayed or poor decision making during critical moments. This can slow the overall response and increase the risk of harm ( Meredith et al., 2011).

The two major constructs in developing effective intervention strategies in disaster risk reduction include recognizing the profound impact of hazard characteristics and psychological constructs, as well as understanding them while highlighting psychological preparedness intentions at the center of disaster management programs. In summary, improving individual performance and reducing the total damage caused by disasters require incorporating psychological preparedness into disaster readiness. This entails understanding how psychological characteristics and readiness interact, which is crucial for creating successful disaster-management plans.

### Sustainable development goals

The Sustainable Development Goal (SDG) 3, which focuses on good health and well-being, has highlighted the importance of mental health and resilience in addressing resilience during adverse events. The key component of SDG 3, the target of which is to strengthen the prevention and treatment of substance abuse, including narcotic drug abuse and the harmful use of alcohol, can be included to improve psychological preparedness and resilience. Disasters may also lead to increased substance use ( Bonanno et al., 2010). Therefore, cultivating psychological preparedness and resilience towards inevitable disasters could be a point to reach SDG 3 in the future.

Additionally, SDG 13, which is Climate Action, could also be relevant, especially because this study addresses climate-related crises. In this case, in Malaysia, psychological preparedness is essential for adaptation. Emphasizing climate resilience while connecting psychological preparedness to the increasing frequency of climate-induced crises such as floods could help support broader efforts to strengthen community resilience.

## Literature review

### Evolution of preparedness towards flood in malaysia

The National Disaster Relief Committee was founded in 1972 in Malaysia following the flood of 1971 and is responsible for flood mitigation, prevention of fatalities, and organizing relief efforts at the federal, state, and local levels ( Shah et al., 2017). During floods, a Short Message System (SMS) notifies important government institutions, and public and government agencies may obtain real-time rainfall and river level statistics on the Public Infobanjir (FloodInfo) webpage ( Khalid & Shafiai, 2015). Emergency reactions can be handled quickly and effectively owing to this readiness.

As Malaysia’s economy and industry grow, there are also emergency protocols for disasters. Disaster relief activities were governed by National Security Council Directive 20, which came into force on May 11, 1997. These include large industrial mishaps, airplane accidents, and natural disasters. Its goal is to put in place an all-encompassing emergency management system to lessen the effects, handle emergencies, and deliver assistance. Organizations that provided assistance included the Red Crescent and St. John Ambulance, the Armed Forces, SMART, the Fire and Rescue Department, the Royal Malaysian Police, and emergency medical services ( Aini et al., 2001). These steps guarantee effective and coordinated flood control at the level of physical preparedness.

### Different areas of preparedness towards floods

Increasing knowledge and readiness among stakeholders exposed to natural disasters is the primary objective of flood preparedness. Plans or preparations must be made to assist in response and rescue service activities and safeguard people’s lives or property. Building capacity, implementing an early warning system, and maintaining flawless operations are necessary to guarantee that the public can react appropriately to warnings. The following section provides further details of Malaysia’s flood preparedness programs, as reported by Noorhashirin et al. (2016).

The Department of Irrigation and Drainage (DID), the Malaysian Meteorological Department (MMD), and the Malaysian Remote Sensing Agency (MRSA) are important organizations engaged in flood preparedness in Malaysia. The early warning systems and emergency evacuations due to severe weather and wind were managed by the DID. Furthermore, MRSA delivers cutting-edge remote sensing technology essential for agriculture, resources, the environment, disaster management, security, and land development, while MMD provides weather reports and flood forecasts via alerts ( Noorhashirin et al., 2016). Noorhashirin et al. (2016) proposed that SMS alerts be distributed to the general public to improve community communication about disasters. Now, an app called MyPublic Infobanjir has also been developed to provide an alert notification to the public, internal users of the Department of Irrigation and Drainage (DID) Malaysia, and external agencies for the dissemination of flood forecasts and warnings.

Additionally, the Infobanjir portal provides direct access to real-time rainfall and river water level data that are published online by the general public and government personnel. The public may effectively receive early flood warnings via the public Infobanjir system at any time and from any location that has internet connectivity. After the devastating floods of December 2014, the government took proactive steps to better manage future floods. These included setting up an early warning system and allocating RM700 million for flood mitigation projects in Terengganu and Pahang ( Azlee, 2015). By doing so, people can receive real-time updates on water levels and preparedness prior to impending flooding.

Despite the availability of several flood prediction methods and advanced equipment (such as weather radar and satellites), the evacuation process has moved slowly. Numerous people have been stranded by rapidly increasing water levels, but emergency operations are hampered by the locals’ unwillingness to leave their homes and belongings. Aid delivery is complicated since many residents, in spite of the warning signs, choose not to evacuate because they believe floods are minimal ( Noorhashirin et al., 2016). To minimize casualties and damage, effective preparedness necessitates both government action and community readiness for prompt and smooth evacuation.

Several structural solutions have been put in place by the Department of Irrigation and Drainage Malaysia (DID), including flood control dams, river canalization, river bunding, storage ponds for flood attenuation, and flood diversion routes or tunnels. Furthermore, the DID, the Ministry of Education, the Ministry of Health, and other organizations have implemented non-structural measures such as population relocation, individual home floodproofing, and the establishment of guidelines and design standards ( Abdullah, 2017).

A game-based strategy was created to improve youth flood preparedness, which included a board game with question cards to gauge awareness levels before and after exposure, as well as an e-book on the subject ( Ridzuan et al., 2023). Participants gained an understanding of how to safely evacuate during floods, prepare for floods, and recover from damage after using these techniques. Interactive tests, escape games, and real-time access to flood management websites, such as National Disaster Management Agency (NADMA) and InfoBanjir, are other features of a flood preparedness module in a metaverse setting ( Sa Don et al., 2023). According to Noorhashirin et al. (2016), these steps are intended to lessen the destructive effects of floods and ensure that knowledge about flood hazards is extensively shared.

### Psychological impact of natural disasters

Posttraumatic Stress Disorder (PTSD), sadness, anxiety, and even suicide are among the psychological effects of natural catastrophes on human resources ( Amiri & Jahanitabesh, 2022). Survivors and first responders who took part in search and rescue operations are also among the people affected by the post-disaster effect, which can have long-term psychological effects on them ( Morgan, 2016; Novia et al., 2020). Research on disaster mental health revealed that survivors of natural disasters encounter difficulties in adjusting to the aftermath, and these events’ emotional toll has a significant negative impact on their well-being, necessitating constant attention.

It was discovered that, among the student population, the trauma of an unforeseen disaster led to mental health issues ( Kato et al., 2017). In addition, some individuals are reluctant to return to school because they are afraid of local natural disasters ( Magni et al., 2017). PTSD, depression, and anxiety can emerge following disasters and have been linked to significant morbidity and mortality. Therefore, early diagnosis and evidence-based therapies are necessary ( Morganstein & Ursano, 2020). Research conducted following the Wenchuan earthquake in China in 2008 revealed that depressive symptoms, PTSD, and sleep disturbances were common and lasted for 12 and 24 months in teenagers ( Fan et al., 2017). This shows that the psychological impact can be prolonged up to two years after the tragedy.

This idea was supported by another study on flood victims in Indonesia; its results showed that 52% of the respondents who were impacted by flooding had PTSD. According to Nasri et al. (2020), the majority of respondents reported having re-experienced symptoms (98.3%), which entailed having recurring memories of the incident. This illustrates how victims and first responders who observed a terrible event could experience mental health consequences as a result of natural disasters.

Psychological preparedness plays a crucial role in mitigating the negative mental health consequences of natural disasters. Individuals with higher levels of disaster preparedness exhibited fewer symptoms of depression and anxiety. A study following the Türkiye earthquakes also found that those directly affected had significantly lower preparedness and higher mental health issues ( Öztekin & Örki, 2023). Therefore, strengthening resilience and disaster prevention capacity, while improving disaster preparedness, is an effective strategy to deal with disaster risks and improve residents’ well-being ( Ma et al., 2021).

### Psychological preparedness

According to Reser and Morrissey (2009), psychological preparedness is different from physical or household preparedness, and refers to a person’s readiness, expectation, and awareness. It entails managing one’s emotions in emergency situations, lowering the danger of a disaster, and adjusting to natural calamities ( Paton, 2020). When people are psychologically prepared, they can remain calm and compose in the face of calamity, allowing them to help others more successfully. According to Reser and Morrissey (2009), preparedness reduces catastrophic injuries and fatalities.

In times of high and prolonged stress, negative emotions such as fear and anxiety disrupt one’s working memory and may cause permanent dysregulation of neurobiological stress systems ( Khayyer et al., 2017; McEwen et al., 2015). However, in situations of an acute challenge, the stress response can be a healthy and adaptive function ( Engert et al., 2019). Stress has also been linked to a number of cognitive performance problems, including the perception of danger, decision-making, attention, memory, and focus. These problems may have an impact on an individual’s behavior ( Helton et al., 2011; Reser, 1996; Starcke et al., 2008).

Malaysian communities exhibit a multifaceted perception of and response to disasters, particularly floods, shaped by local knowledge, social vulnerability, and resilience frameworks. These communities actively engage in disaster risk reduction (DRR) strategies, leveraging both traditional practices and modern frameworks to enhance their flood preparedness and response capabilities. Urban communities, such as those in Bukit Antarabangsa, recognize their social vulnerability to landslides and floods, leading to community-based disaster risk reduction initiatives ( Kamarudin et al., 2022). Research has reported that residents are aware of their risks and actively participate in discussions on vulnerability and resilience strategies. The establishment of Community Resilience Frameworks is essential for guiding stakeholders in building disaster-resilient communities ( Sulaiman et al., 2019). At the state level, Malacca plays an active role in resilient city development. The Malacca State Government has introduced city-resilient initiatives with various strategies, such as disaster risk reduction programs, local councils for sustainability, 100 resilient cities, and local plans and policies ( Jamaludin & Sulaiman, 2018). Conversely, although communities are proactive in disaster management, challenges remain, particularly in integrating scientific knowledge with local practices and ensuring equitable access to resources for all community members. This highlights the need for continuous improvements in disaster preparedness strategies.

This problem has negative effects such as narrowing attention to only a few aspects of the situation, omitting threats, taking longer than expected to complete tasks, having trouble remembering crucial information, and making it difficult to recall survival-related details ( McLennan et al., 2011). As a result, to spur protective activities during flood preparedness measures, a certain amount of stress is necessary, but too much stress will have a detrimental effect on flood preparedness actions during these crucial moments. One aspect of psychological preparedness for natural disasters is the capacity to control emotions and build resilience in those who are impacted.

**Figure 1.**
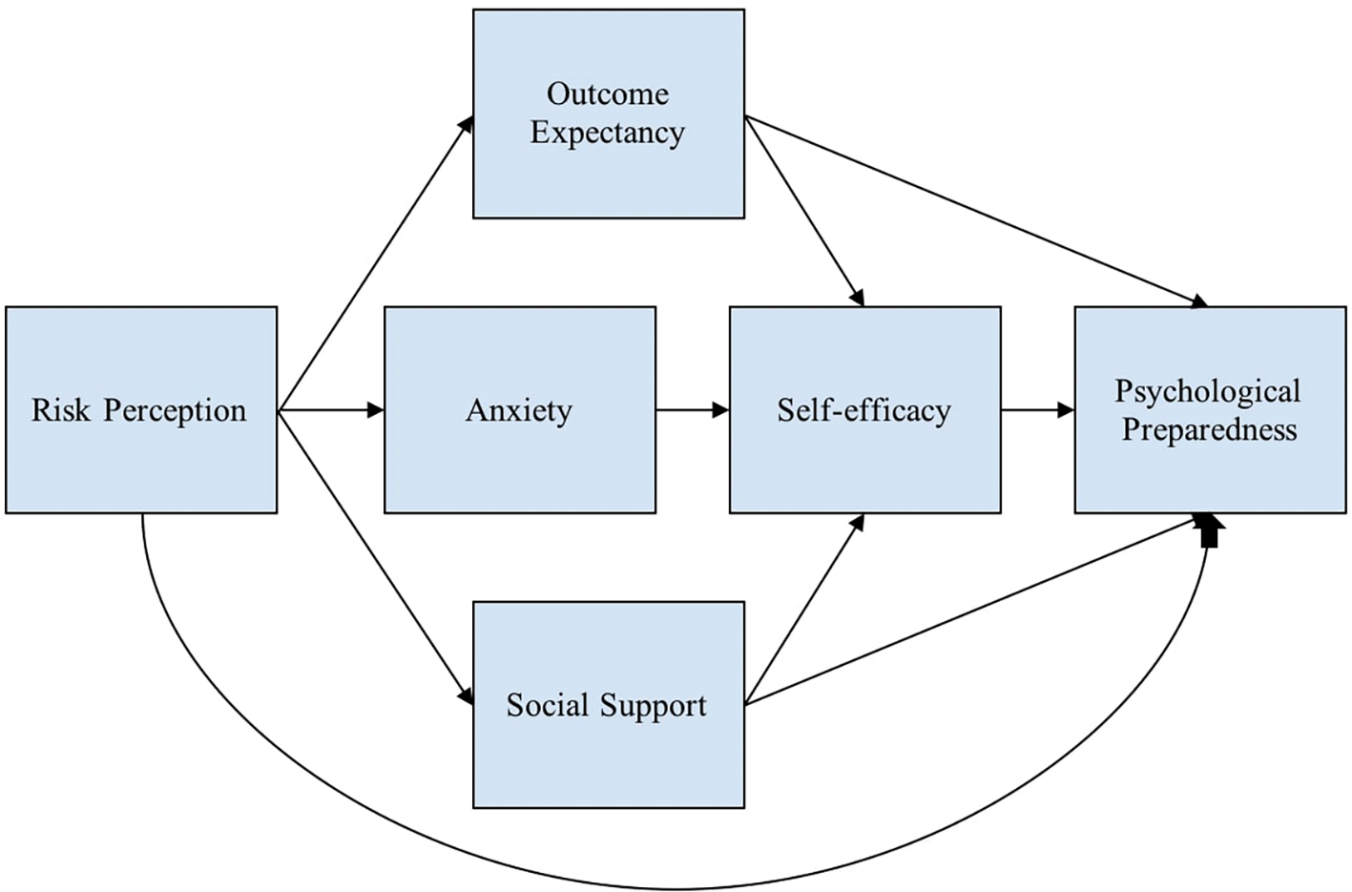
Conceptual framework. Note: This modified figure from Paton (2003) is used to describe the variables that predict psychological preparedness.

Research shows that people with higher levels of stress, anxiety, and depression are less prepared for disasters, highlighting the importance of mental health interventions before disasters ( Öztekin & Örki, 2023). The results showed that the participants’ sociodemographic traits differed significantly in terms of their stress levels, anxiety symptoms, and depression symptoms. Gender, age, education level, marital status, and personal experience with the February 6 earthquake in Turkey were the main factors influencing disaster preparedness. The following conceptual framework is proposed based on insights from previous studies. Figure 1 shows the conceptual framework used to describe the variables that predict psychological preparedness.

### Psychological preparedness towards disaster

Better disaster preparedness efforts are a result of factors such as place reliance, threat perception, and disaster likelihood, as demonstrated by the landslides that occur among farming households in China ( Xu et al., 2018). Nevertheless, a different study conducted on homeowners in Cologne, Germany, who lived in flood-prone areas, found no correlation between taking preventative measures and fear of future flooding ( Grothmann & Reusswig, 2006). This paradoxical conclusion highlights that while risk perception may encourage people to take proactive preparation actions by evaluating hazards, this is not always the case. In addition, individual experiences with natural catastrophes and confidence in authorities are among the factors that influence how people perceive risk and prompt them to take preventive measures ( Wachinger et al., 2012). In conclusion, perceptions of risk can be greatly influenced by recognizing and comprehending the possibility and threat of natural disasters, as well as by individual experiences and faith in authority. These factors might ultimately lead people to adopt preparedness behaviors.

### The role of outcome expectancy, self-efficacy and anxiety towards disaster preparedness

Outcome expectancy also plays a crucial role in disaster preparedness. The notion of whether one’s own activities will successfully lessen or minimize a problem is known as outcome expectancy ( Paton, 2003). According to Johnston et al. (2005), non-preparation is caused by negative outcome anticipation, whereas positive outcome expectancy has a direct impact on both readiness intention and actual preparedness. In a different study, it was shown that residents’ perceptions of “dragons of inaction,” or potential obstacles to their intentions to prepare, were stronger when they had stronger positive outcome expectancy beliefs. Although there is no direct correlation between positive outcome expectancy and preparedness, it is significantly mediated by dragons of inaction ( Asgarizadeh & Gifford, 2021). This demonstrates that there is an indirect, rather than a direct, association between outcomeexpectancy and disaster preparedness.

Previous experience with flood disasters influences various psychological aspects, such as positive outcome expectancy, negative feelings, perceived likelihood, perceived consequences, anxiety, trust, preparedness intention, and community preparedness for flood hazards ( Ejeta et al., 2018). Specifically, negative outcome expectancy, a sense of personal responsibility for preparedness, and children engaging in hazard education programs have emerged as key predictors of preparedness related to natural disasters ( Kelly & Ronan, 2018). Despite these insights, there remains a gap in the research addressing the role of outcome expectancy in relation to psychological preparedness during natural disasters. Prior research suggests that expectancies represent indirect inductions that indicate the likely anticipated outcome (i.e., this is likely to happen), but still require the participants to generate a final response to prepare for that likely outcome (that is, given this is likely to happen, I will respond this way. Therefore, there are multifaceted impacts on psychological factors and community preparedness; however, the significance of outcome expectancy in the context of psychological preparedness for natural disasters requires further exploration because of the limited number of existing studies.

Self-efficacy has been highlighted as a key variable in disaster preparedness. Self-efficacy refers to a person’s confidence in their ability to perform a specific behavior in the face of various obstacles ( Glanz et al., 2008). Moreover, in situations involving natural disasters, self-efficacy is a prerequisite for the adoption of resilience and adjustment adoption ( Floyd et al., 2000; Neuwirth et al., 2000). Self-efficacy has a significant impact on the quantity and calibration of actions taken, as well as the persistence and effort put forth in risk mitigation ( Paton, 2003). People are more likely to participate in preparedness activities when they feel more self-assured or self-efficacious about their capacity to handle a particular scenario such as an emergency ( Paek et al., 2010). Consequently, self-efficacy affects disaster preparedness.

Although anxiety has been associated with worry, it also propels disaster preparedness. In the Indian population, anxiety has been associated with decreased preparedness for heat waves and floods ( Mishra & Suar, 2012). Being ready for future disasters may lessen anxiety and depressive symptoms, which would improve mental health in general (e.g. Galappatti & Richardson, 2016). Better preparedness may stem from better mental health, and preparedness practices may have a favorable effect on mental health. To the extent that preparedness minimizes the impact of future disasters, it also protects against the linked mental health implications of looming disasters ( James et al., 2019).

### Risk perception, outcome expectancies, anxiety, social support and self-efficacy

Perceiving risk and outcome expectancies could be the first step towards preparing to face disasters. In a 2018 study on flood preparedness in rural families, Mabuku et al. (2018) discovered that worry and risk perception had an impact on flood preparedness. It was also discovered that a high degree of flood preparedness is linked to positive outcome expectancy and risk perception or worry about risk. A qualitative interview by Becker et al. (2013) revealed that recognizing the limitations of preparedness does not automatically lead to negative outcome expectations, but the benefits of preparation must be seen to balance the limitations.

This can be explained by the fact that people’s negative outcome expectation beliefs are supported by the uncertainty surrounding the nature of hazard events and their possible repercussions. Stated differently, a hazy understanding of risk may cause people to anticipate unfavorable results in the event of a crisis. Risk perception and anxiety were also associated with disaster preparedness. Various studies have shown that people experience unpleasant emotions in response to calamities. For instance, Altarawneh et al. (2018) revealed that negative emotions (distress, anxiety, wrath, and powerlessness) were significantly predicted by the sense of flood risk. When the students participating in this research thought that the landslide was uncontrollable, and when they feared the landslide, their thoughts on the possibility of landslides increased ( Peng et al., 2017). The anxiety of students grows when they believe that they will encounter calamities, be impacted by them, and suffer from damage.

According to this study, pupils who were more exposed to disasters were considered to have a significant impact, thought they were uncontrollable, and had higher anxiety levels ( Mızrak & Aslan, 2020). Consequently, it is possible to infer that anxiety about natural disasters is related to risk perception.

Social support has been shown to assist communities in disasters. Although social support has been extensively studied in post-disaster response and recovery ( Aldrich, 2012; Bolin, 1982; Kaniasty et al., 1990; Mutch, 2015), it has not been extensively studied in the literature on disaster preparedness ( Reininger et al., 2013). Nevertheless, the best indicator of pre-earthquake preparedness in a study of families conducted before and after earthquakes was social support provided to others ( Heller et al., 2005). However, according to another study, reciprocity and group participation were not linked to the adoption of preparedness measures ( Reininger et al., 2013). As the idea of belonging is a fundamental human motivation ( Baumeister & Leary, 1995), the role of social support from both formal and informal channels should be investigated to understand and facilitate the public’s preparedness for disasters.

According to Han et al.’s study from 2021, people who had greater informal social support, including personal networks, had a lower sense of risk and were less likely to take formal support from formal organizations during emergency preparedness. These findings suggest that, as shown in Austria, the expectation of social support may minimize perceived dangers and reduce the likelihood that an individual will take preventative measures ( Babcicky & Seebauer, 2017). The disparities between the effects of formal and informal social support may suggest that informal personal networks’ social support is more important than formal organizations’ social support in predicting people’s perceptions of the risks associated with natural disasters or their preparations for disasters.

People with high-risk perception may not know what to do when it comes to preparation, which is one of the main reasons for not taking preparedness measures ( Wachinger et al. 2012). While response knowledge can influence self-efficacy and response efficacy because it can result from possessing the knowledge necessary to reduce risks, it can influence how risks are perceived ( Lindell & Whitney, 2000). A previous article stated that the focus of research and practice on disaster risk reduction and climate adaptation may need to shift from risk perception and climate change perception to social norms and efficacy. The results showed that adaptive behaviors were positively predicted by perceptions of social norms and self-efficacy ( Lim, 2022). Therefore, one can effectively prepare for approaching disasters before they occur by having knowledge and identifying the risk.

### Outcome expectancy, self-efficacy and psychological preparedness

According to Samaddar et al. (2014), outcome expectations and self-efficacy expectations are indicators of one’s beliefs regarding one’s own talents and action-outcome links. This can be applied during crucial times, such as during natural disasters. According to Grothmann and Reusswig (2006), intention to implement flood prevention measures is positively correlated with both self-efficacy and outcome expectancy. Additionally, according to the conventional cognitive theory of risk-mitigation behaviors, an individual’s motivation to protect themselves is thought to occur in stages. Specifically, after they believe that a hazard will negatively impact them, they begin to consider the potential benefits of taking action (called outcome-expectancy) and question their ability to carry it out (called self-efficacy) ( Duval & Mulilis, 1999; Grothmann & Reusswig, 2006; Martin et al., 2007; Neuwrith et al., 2000; Rogers, 1983). According to Samaddar et al. (2014), there is a significant positive association between self-efficacy and outcome expectancy among Mumbai’s slum populations. For instance, a disaster victim might evaluate his or her abilities, resources, and knowledge to put a specific disaster preparedness measure into practice if they believe it to be effective (high positive outcome-expectancy). Based on this evaluation, disaster preparedness intentions can then be developed ( Strecher et al., 1986).

The process from outcome expectancy to psychological preparedness can be explored through the mediating role of self-efficacy. Grothmann and Reusswig (2006) demonstrated that both conceptions of self-efficacy and outcome expectancy are positively connected to the adoption intention of flood preventative measures. It has been demonstrated that self-efficacy may play a mediating role in the relationship between psychological preparedness and outcome expectancy. A Chinese study discovered that self-efficacy mediates the relationship between readiness for disasters and place attachment, emphasizing the significance of boosting self-efficacy in disaster preparedness campaigns ( Wang et al., 2021). According to a different US study, people are more likely to take readiness actions when they have higher levels of self-efficacy, which clarifies the cognitive processes underlying preparedness behaviors ( Miao & Zhang, 2023). People’s self-efficacy increases when they think that taking proactive measures would pay off. Greater psychological preparedness, as evidenced by mental readiness and confidence in handling disaster-related issues, is a result of elevated self-efficacy.

### Risk perception, outcome expectancy, social support, self-efficacy and psychological preparedness

Self-efficacy can also act as a mediator of the relationship between psychological preparedness and risk perception. When people believe there is a significant chance of a calamity, such as flooding, they are driven to evaluate and improve their capacity to contain the threat. Research on flood risk showed that people take adaptation measures to shield themselves from floods if they perceive the risk of the hazard they face to be high and if they think the protective measures that are available are relatively easy (high self-efficacy) ( Poussin et al., 2015).

Self-efficacy, or a person’s belief in their own ability to handle an emergency or natural disaster, is one of the wellbeing-related variables that can support resilience ( Babcicky & Seebauer, 2017; Hudson et al., 2019). According to Benight et al. (1999), individuals who had higher levels of self-efficacy also reported higher levels of subjective well-being and were better equipped to handle the stress that comes with “natural disasters.” Individual self-efficacy has also been found to be positively connected with disaster preparedness ( Adams et al., 2019) and a propensity for active behavior when coping with stress ( Chudzicka-Czupała & Zalewska-Łunkiewicz, 2020). Consequently, risk perception encourages self-protection through self-efficacy, which fosters psychological preparedness for emergencies.

People who live in a flood-prone area and perceive a high likelihood of flooding are likely to assess their level of preparedness and look for ways to improve. Workshops can be a useful method for communicating risks effectively, increasing awareness, and encouraging precautionary behavior among people living in flood-prone areas ( Heidenrich et al., 2020). As they acquire knowledge and skills, their self-efficacy increases, leading to an increase in their psychological preparedness. This enhanced confidence in their ability to respond appropriately during a flood reduces their anxiety and boosts their overall resilience. Consequently, because they were already aware of the danger of a disaster, victims who had personally experienced flooding felt more confident about their ability to prevent future floods ( Ogunbode et al., 2018).

The transition from psychological preparedness to risk perception can be understood by considering the mediating functions of outcome expectancy and self-efficacy. People assess the possible consequences of their preparedness measures when they believe there is a high likelihood of a disaster such as a flood. Additionally, it has been shown that the intention to take flood prevention measures is positively correlated with outcome expectancy and self-efficacy ( Grothmann & Reusswig, 2006). The conceptualization model of risk behavior shows that risk perception is a dominant characteristic of individual risk behavior ( Mendis & Peter, 2020). The desire to be ready for danger is directly and favorably influenced by one’s sense of risk ( Pinto et al., 2023).

This implies that the relationship between risk perception and preparedness intention may be mediated by outcome expectancy. Moreover, it has been found that perceived coping efficacy has a mediating role in risk perception and disaster preparedness ( Miao & Zhang, 2023). Since self-efficacy has been found to be a strong predictor of preparedness behaviors, more self-efficacy can also translate into greater psychological preparedness, which is characterized by mental readiness and confidence in addressing issues related to disasters ( Bourque et al., 2013).

Thus, it may be possible to encourage the best possible engagement in preventive behaviors by comprehending the mediating roles that outcome expectancy and self-efficacy play in the pathway from risk perception to psychological preparedness ( Tagini et al., 2021). These elements can guide tactics and interventions to encourage readiness in the face of diverse hazards. For example, people who live in areas that often experience flooding and who think there is a high chance of flooding might first think that taking certain precautions, such as making plans for evacuation and constructing barriers, will reduce the likelihood of harm. This positive outcome expectancy enhances their self-efficacy, making them feel capable of efficiently completing these tasks. Consequently, they become more psychologically prepared, making it easier for them to deal with the pressures and demands of a flood.

Self-efficacy and social support provide an avenue for understanding their mediating roles in risk perception and psychological preparedness. People look for social support when they believe there is a high chance of a disaster, such as flooding, in order to help them get prepared. It has been discovered that social support, particularly subjective support and support utilization, favorably affects self-efficacy, which in turn improves psychological outcomes and self-management in people who are ill ( Al-Dwaikat et al., 2021). However, as a cognitive psychology term, general self-efficacy also has a major impact on people’s actions toward achieving specific goals ( Mendis & Peter, 2020). Social support provides them with the tools, knowledge, and emotional support they need to increase their sense of self-efficacy.

### Role of anxiety, self-efficacy and risk perception in psychological preparedness

How is anxiety related to psychological preparedness? According to Lazarus (1991), those with dispositional anxiety tend to view every circumstance as threatening, whereas those with high trait anxiety scores are more likely to adapt to disasters. Trait anxiety has been shown to play a role in psychological preparedness for disasters (Malkina-Pykh et al., 2013). According to Elwood et al. (2012), trait anxiety is the propensity of a person to perceive situations as dangerous, steer clear of anxiety-inducing circumstances, and exhibit high baseline physiological arousal. The connection between anxiety and actions related to catastrophe preparedness is complex. Anxiety and health-promoting behavior, as well as preparedness towards terrorist attacks, have been shown to have a negative relationship in the past ( Wirtz et al., 2019).

In another study in the workplace context, employees’ anxiety levels and preparedness for earthquake emergencies revealed a favorable correlation between preparedness and anxiety levels ( Surbariyanti et al., 2019). These studies highlighted inconsistent findings, such as the negative association between anxiety and readiness for nurses, and demonstrated the complex relationship between anxiety and disaster preparedness ( Said et al., 2020; Wirtz et al., 2019). There was a noticeable gap between trait anxiety and psychological preparedness in terms of the impact of anxiety on both psychological and physical preparedness.

Wirtz et al. (2019) in their study showed that anxiety was negatively related to self-efficacy in the context of disaster preparedness. Specifically, anxiety has been found to have a significant influence on disaster preparedness, with higher anxiety levels associated with lower levels of preparedness ( Öztekin & Örki, 2023). In a study on individuals with physical disabilities who faced the threat of human-made and natural disasters, individuals who reported an increase in self-efficacy also reported a decrease in psychological distress ( Reed et al., 2023). In summary, there was a negative association between anxiety and self-efficacy during natural disasters, which highlights the importance of addressing anxiety to improve disaster preparedness and reduce psychological distress.

Previous studies have identified a negative relationship between anxiety and health-promoting behaviors, suggesting that anxiety can impact disaster preparedness behaviors for disasters ( Wirtz et al., 2019). Previous studies have demonstrated the role of self-efficacy in mediating disaster preparedness ( Malkina-Pykh & Pykh, 2013; Wang et al., 2021). To explain the psychological mechanisms through which self-efficacy mediates the relationship between anxiety and psychological preparedness, it can be deduced that anxiety operates on preparedness behaviors through multiple pathways, such as perceived threat and self-efficacy ( Wirtz et al., 2019). Additionally, self-efficacy mediates the relationship between place attachment and disaster preparedness, highlighting the importance of promoting self-efficacy in disaster preparedness efforts ( Wang et al., 2021). In summary, anxiety negatively affects psychological preparedness and can lead to increased worry, affecting individuals’ readiness for natural hazards. However, self-efficacy still plays a crucial mediating role between anxiety and psychological preparedness, highlighting the importance of promoting self-efficacy in disaster preparedness.

The pathway from risk perception to psychological preparedness for natural disasters can be understood through the mediating roles of anxiety and self-efficacy. Risk perception plays a crucial role in predicting anxiety in natural disaster situations, and studies have shown that direct experiences, such as material and emotional consequences of an earthquake, indirectly influence risk perception through worry ( Bronfman et al., 2020). This showed that when individuals perceive a high risk of a disaster, such as a flood, they often experience anxiety about its potential impact. This anxiety motivated them to evaluate their ability to manage threats. If they believe that they can take effective action, their anxiety is channelled into a sense of control, enhancing their self-efficacy. One study found that self-efficacy plays a role in undertaking preparedness measures, whereby it increases individuals’ propensity to engage in disaster preparedness behaviors ( Miao & Zhang, 2023). Disaster experiences have also played a role in experiencing a disaster in the past five years and were found to increase self-efficacy in undertaking preparedness measures and perceived efficacy of preparedness ( Miao & Zhang, 2023). Another study on preparedness for terrorist attacks found that anxiety operated on preparedness behaviors through multiple pathways, including perceived threat, which is a part of risk perception and self-efficacy ( Wirtz et al., 2019). Longitudinal testing revealed that an increase in self-efficacy is a way to reduce disaster-related psychological distress ( Reed et al., 2023). These findings suggest that both anxiety and self-efficacy play crucial roles in influencing individuals’ preparedness behaviors, highlighting the complex interplay of cognitive and affective factors in disaster preparedness.

### Social support, self-efficacy and psychological preparedness

Social support has been shown to have a negative effect of traumatic events on depressive and PTSD symptoms among individuals affected by Hurricane Katrina ( McGuire et al., 2018). Additionally, informal social support has been found to positively affect disaster preparedness, with perceived collective efficacy and self-efficacy playing mediating roles in individuals’ disaster preparation ( Yu et al., 2022). A study on disaster response following Hurricane Matthew revealed that self-efficacy significantly affects engagement in protective behaviors, emphasizing the psychological mechanisms through which self-efficacy, influenced by social support, affects disaster response ( Wong-Parodi & Feygina, 2018). This indicates that social support plays a crucial role in predicting self-efficacy during natural disasters. Emotional, informational, and practical support from social networks provides the necessary resources, encouragement, and knowledge that enhance individuals’ confidence in their ability to prepare for and respond to disasters. Understanding the mechanisms through which social support influences self-efficacy can inform the design of disaster preparedness programs that leverage community strength to build resilience and ensure effective disaster management.

Self-efficacy reflects one’s perception of their capacity to conduct an action, and outcome efficacy is the measurement of the perceptions of necessary actions in reducing a problem ( Malkina-Pykh & Pykh, 2013). According to Gandhi et al. (2021), self-efficacy, optimism, and resilience have emerged as predictors of psychological preparedness. Multiple linear regression analysis revealed that self-efficacy, optimism, and resilience explained 62% of the variance in psychological preparedness. Self-efficacy has been linked to better psychological functioning ( Gallagher et al., 2002). Additionally, in the context of frontliners, high self-efficacy perception contributes to better intervention and effective performance of nurses in the event of a disaster ( Kılıça and Şimşek 2019). Additionally, a study conducted in Indonesia by Fa’uni and Diana (2021) revealed a significant relationship between self-efficacy and psychological preparedness for disasters. Therefore, self-efficacy plays a role in predicting psychological preparedness for natural disasters, such as floods.

Several studies have highlighted the mediating role of self-efficacy on disaster preparedness. For instance, one study found that self-efficacy played a mediating role between place attachment and disaster preparedness ( Wang et al., 2021). Another study demonstrated that self-efficacy mediates the relationship between received social support and coping self-efficacy, ultimately mitigating psychopathology risk among healthcare workers during the COVID-19 pandemic ( Smith et al., 2022). The findings suggest that social support and self-efficacy are correlated with higher degrees of overall preparedness and specific preparedness behaviors ( Wang et al., 2021). Additionally, the importance of promoting self-efficacy and hope in earthquake survivors, along with the necessity of social support in reducing stress factors caused by earthquakes, has been highlighted ( Ime, 2024). Perceived collective efficacy and self-efficacy have been identified as mediators that differ in their effects on people’s disaster preparations, with collective efficacy being effective in stimulating individual preparation in slow-onset disaster environments and self-efficacy being effective for sudden onset disaster environments ( Yu et al., 2022). When individuals receive social support, their confidence in their ability to manage disaster-related tasks is increased. This enhanced self-efficacy leads to greater psychological preparedness, characterized by mental readiness, emotional stability, and confidence in handling disaster challenges.

## Methods

To investigate the psychological preparedness towards flood, a systematic literature review was conducted to investigate the psychological preparedness towards flood. Databases such as PubMed, Academic Search Ultimate, Directory of Open Access Journals (DOAJ), Supplemental Index and Google Scholar were used to search for relevant articles. Keywords employed include “disaster preparedness”, “flood”, “natural disaster” and “psychological preparedness”. Boolean operators (AND/OR) were used to refine the search engine for better results. The articles were included in the review if they focused on preparedness towards disasters as well as examined the coping mechanisms, perceptions, or preparedness behaviours in the context of flood-related crises. Articles will be excluded if they did not meet the required methodological standards and were outside of the scope of crises.

To synthesize the findings, a thematic analysis approach was utilised. The key themes and subthemes related to psychological preparedness, coping strategies and mental health implications were highlighted. A conceptual model describing the factors predicting Malaysians’ psychological preparedness for floods was developed using these findings as a guidance.

The proposed conceptual framework was developed based on the synthesized findings and insights from current theories on catastrophic risk reduction and psychological resilience which served as the foundation for the framework development. The framework aims to direct future research and improve disaster management strategies by emphasising the critical variables in enhancing psychological preparedness.

### Importance of psychological preparedness in disaster management

Psychological preparedness as a part of physical readiness is of primary importance, as it not only influences physical response during different situations leading to actions but also serves as a primary element in reducing the physical, social, psychological, and sociocultural harm of disaster, which causes people to cope with disasters and derive a sense of safety ( Blake et al., 2017; Margaritova, 2008). Psychological preparedness enables individuals to perform properly and promptly during a disaster happens ( Reser & Morrissey, 2009; Suhaimi & Marzuki, 2016). Thus, the sum of internal awareness, readiness, and anticipation is intricately shaped by personality traits such as anxiety, self-efficacy, optimism, and self-esteem ( Malkina-Pykh & Pykh, 2013). The two major constructs in developing effective intervention strategies in disaster risk reduction include recognizing the profound impact of hazard characteristics and psychological constructs, as well as understanding them while highlighting psychological preparedness intentions at the center of disaster management programs. In conclusion, integrating psychological preparedness into disaster readiness is essential to enhance individual performance and reduce the overall harm caused by disasters. This involves recognizing the interplay between psychological traits and preparedness, which is central to developing effective disaster management strategies. 

### Application of the proposed model

As such, due to the limited number of studies on psychological preparedness towards floods in Malaysia, an exploratory study could be utilized. Future research could employ mixed methods because qualitative insights can deepen the understanding of individual psychological responses obtained through interviews or focus groups, while quantitative methods such as surveys can statistically assess the relationships between variables such as risk perception, self-efficacy, and psychological preparedness.

Although the suggested model is supported by research evidence, it can be tested using surveys conducted among flood victims. Additionally, interviews or focus groups could explore lived experiences and validate the interaction between these factors, particularly in Malaysia’s cultural context. Structural equation modelling (SEM) can be used to analyze the relationships between the constructs in the conceptual model. Qualitative analysis methods, such as thematic analysis, can be used to explore the interviews in exploring emergent themes around preparedness.

Psychological preparation plays a role in mitigating risks, but the subject of psychological preparedness still appears to be an under-researched area ( Boylan & Lawrence, 2020). Although floods are frequent natural disasters in Malaysia, academic literature on flood preparedness is still scarce ( Sa don et al., 2023). Although some studies have examined the material aspects of disaster preparedness responses, psychological dimensions are not being given enough attention which includes emotional reactions, coping strategies, and utilization of existing resources ( Diyana et al., 2020; Jani et al., 2015). A previous study on the psychological functioning of flood-affected girls also revealed that those who received prevention of posttraumatic stress reactions had better psychological functioning ( Dildar & Kausar, 2019). The argument now is that adequate preparation can make it possible to significantly reduce the impacts of flood disasters through a good understanding of preventive action, as well as knowledge of some life-saving techniques during disasters ( Ibem, 2011). Neglecting psychological preparedness can result in long-term mental health issues, such as PTSD, depression, and anxiety disorders, among affected populations. This can strain healthcare systems and hinder overall recovery efforts ( WHO 2023). Nowhere has the issue of floods become a developmental issue than in poor and developing countries where systemic problems and institutional constraints have increased vulnerability (social, economic and physical) to flood risk and thus, reducing resilience to flood disasters.

This article builds on and contributes to addressing the research gap by exploring the factors associated with psychological preparedness among Malaysians facing a flood disaster to provide insights into the experiences and emotional responses, coping strategies, and resources during the disaster. By understanding these factors, a more targeted and effective intervention can be developed.

The importance of psychological preparedness is also highlighted by a prior article by Suhaimi and Marzuki (2016), who addressed the significance of psychological preparedness among flood victims, but did not address the current state of affairs. According to Suhaimi and Marzuki (2016), psychological preparedness is one of the most important factors that must be monitored and adjusted before and during a disaster to enhance people’s behavior and psychological response. They demonstrated that since psychological preparedness is defined as an intra-individual and mental state of mindfulness, expectancy and availability differ from physical preparedness. Thus, determining the current psychological preparedness of flood victims is essential in facing disastrous circumstances. Moreover, academics and medical experts in the field of emergency and disaster recovery management should highlight the behavioral change models associated with emergency preparedness ( Samah et al., 2019). Samah et al. (2019) suggested that more empirical research should be conducted to explore the relationships between the models and the benefits of analyzing them.

### Implications

When calamity strikes, those who are psychologically prepared can act appropriately and quickly ( Reser & Morrissey, 2009; Suhaimi & Marzuki, 2016). Accordingly, personality factors, including anxiety, self-efficacy, optimism, and self-esteem, intricately impact total internal awareness, readiness, and anticipation ( Malkina-Pykh & Pykh, 2013). The two major constructs in developing effective intervention strategies in disaster risk reduction include recognizing the profound impact of hazard characteristics and psychological constructs, as well as understanding them while highlighting psychological preparedness intentions at the center of disaster management programs. In conclusion, integrating psychological preparedness into disaster readiness is essential to enhance individual performance and reduce the overall harm caused by disasters. This involves recognizing the interplay between psychological traits and preparedness, which is central to developing effective disaster management strategies among at-risk populations.

The inclusion of psychological preparedness in disaster management plans would have an enormous effect. For instance, community programs that include psychoeducation on catastrophe coping strategies may make vulnerable populations more capable and less nervous. By focusing on building resilience, strength, and optimism, disaster education may improve people’s functional coping skills and effectively lessen the symptoms associated with PTSD reactions ( Hu et al., 2023). Generally, government agencies and mental health professionals may collaborate to provide targeted treatments before, during, and after flood catastrophes. This would reduce posttraumatic stress disorder, encourage greater social bonding, and quicker recovery. By addressing the psychological components of preparedness, Malaysia could reduce the long-term social and economic consequences of flood catastrophes, ultimately saving lives and resources.

### Call-to-action

Disaster management organizations, public health experts, and legislators must prioritize psychological preparedness as a critical element of disaster preparedness because of the increasing frequency and severity of floods in Malaysia caused by the inevitable consequences of climate change. Immediate actions that should be taken include integrating mental health support into existing disaster management frameworks, encouraging community-based resilience projects, and increasing the public understanding of psychological preparedness. Organizations such as the Ministry of Health and the National Disaster Management Agency (NADMA) could take the initiative to incorporate psychological preparedness into disaster risk reduction strategies to ensure that it is not seen as an afterthought, but rather as a crucial part of flood mitigation efforts.

## Conclusion

In Malaysia, psychological preparedness for floods is a crucial but frequently disregarded aspect of disaster management. Although the majority of current techniques have concentrated on logistical and physical components, there is a scarcity of studies exploring individuals’ emotional and cognitive aspects of disaster preparedness. Without a framework, research on the psychological aspects of flood victim management may lack the necessary structure to highlight their importance. This can result in an underemphasis on psychological preparedness compared to material and logistical aspects, leading to incomplete and potentially less effective disaster management strategies. This article highlights a conceptual framework that emphasizes the main elements affecting psychological preparedness, including risk perception, outcome expectancy, self-efficacy, social support, and anxiety. The framework ensures that all critical dimensions, including psychological factinterors, are systematically addressed and integrated into the overall preparedness plan. Policymakers and disaster management organizations may create more comprehensive policies that not only reduce the physical effects of floods but also improve the psychological resilience of impacted people by comprehending how these aspects interact to contribute to the betterment of mental well-being and eventually reach the goal of SDG 3: Good Health and Well-being.

### Ethics and consent

Ethics and consent were not required.

## Data Availability

No data are associated with this article.
