# Supplementary material for: Exploring predictors of psychological preparedness for flood victims: A conceptual framework for Malaysia
Source: F1000Res. 2025 Nov 11;14:195. Originally published 2025 Feb 12. [Version 3] doi: 10.12688/f1000research.160231.3 (PMC12728337; doi:10.12688/f1000research.160231.3)
Supplement: Peer review history

# Reviewer report: Reviewer response for version 3

**DOI:** 10.5256/f1000research.190076.r431838

**Published:** 2025-12-23

**Authors:** Aloysius Gunadi Brata

**Affiliations:** 1 Atma Jaya Yogyakarta University, Depok, Indonesia

I have no further comments to make.

 Best/Alo

Is the work clearly and accurately presented and does it cite the current literature?

Yes

If applicable, is the statistical analysis and its interpretation appropriate?

Not applicable

Are all the source data underlying the results available to ensure full reproducibility?

Yes

Is the study design appropriate and is the work technically sound?

Yes

Are the conclusions drawn adequately supported by the results?

Yes

Are sufficient details of methods and analysis provided to allow replication by others?

Yes

Reviewer Expertise:

-

I confirm that I have read this submission and believe that I have an appropriate level of expertise to confirm that it is of an acceptable scientific standard.

# Reviewer report: Reviewer response for version 3

**DOI:** 10.5256/f1000research.190076.r432409

**Published:** 2025-11-21

**Authors:** Aldani P Wijayanti

**Affiliations:** 1 Universitas Diponegoro, Semarang, Indonesia

Thank you for the opportunity to review this revised version of the manuscript. While the authors have addressed several concerns raised by previous reviewers, there remain significant gaps that need attention, particularly regarding the integration of disaster management frameworks and the depth of psychological preparedness conceptualisation. Below are my focused comments on aspects not yet adequately addressed:

 1. The manuscript fails to explicitly map psychological preparedness factors across the three critical phases of disaster management on recurrent flood case: pre-disaster/preparedness, emergency response, and post-disaster: rehabilitation and reconstruction. The proposed conceptual framework treats psychological preparedness as a static construct rather than a dynamic process that evolves across disaster phases. Additionally, Malaysia experiences recurrent annual flooding, yet the framework does not address how repeated disaster exposure affects psychological preparedness. No discussion of disaster fatigue, habituation to risk, or cumulative trauma effects.

 Recommendation: Restructure the conceptual model to explicitly show which psychological factors are most relevant at each disaster phase, and how these factors interact differently during preparedness, response, and recovery stages. Include discussion of how the framework applies to populations with repeated flood exposure and how psychological preparedness may change over time with experience.

 2. No discussion of how psychological preparedness relates to disaster risk tracking systems/early warning system that already established in Malaysia. The framework does not incorporate how early warning systems and disaster forecasting influence psychological preparedness variables (particularly risk perception and outcome expectancy).

 Recommendation: Include a discussion of how Malaysia’s existing flood monitoring and early warning systems interface with psychological preparedness, and how timely risk information affects the proposed psychological constructs.

 3. The literature review appears to draw heavily from general disaster preparedness literature without sufficient focus on flood-specific psychological preparedness. Limited engagement with literature from Southeast Asian or culturally similar contexts where collectivist values and community-based disaster management are prominent. While the manuscript mentions collectivism briefly, it does not deeply explore how Malaysian cultural values shape psychological preparedness.

 Recommendation: Strengthen the literature review by including more flood-specific psychological preparedness studies, incorporating literature from Southeast Asian disaster contexts (Indonesia, Philippines, Thailand) as long as it is flood-related preparedness, reviewing community-based disaster risk reduction (CBDRR) literature that addresses psychological dimensions. Considerations for cultural adaptation of assessment tools for Malaysian context or proposed measurement approach for the conceptual framework.

 In conceptual studies aimed at building frameworks, particularly within a specific national context such as Malaysia, the function of the literature review extends beyond summarising previous findings. It must critically integrate diverse strands of empirical and theoretical knowledge to justify why certain constructs are appropriate, relevant, and valid for the target population. The conceptual framework must be firmly grounded in flood-specific experiences, culturally situated coping mechanisms, and local disaster governance structures. Unfortunately, the current manuscript falls short in this respect. I recommend the article undergo additional revisions before it is suitable for indexing.

Is the work clearly and accurately presented and does it cite the current literature?

Partly

If applicable, is the statistical analysis and its interpretation appropriate?

Not applicable

Are all the source data underlying the results available to ensure full reproducibility?

No source data required

Is the study design appropriate and is the work technically sound?

Partly

Are the conclusions drawn adequately supported by the results?

Partly

Are sufficient details of methods and analysis provided to allow replication by others?

No

Reviewer Expertise:

Disaster Mental Health, Mental Health and Psychosocial Services (MHPSS)

I confirm that I have read this submission and believe that I have an appropriate level of expertise to confirm that it is of an acceptable scientific standard, however I have significant reservations, as outlined above.

# Reviewer report: Reviewer response for version 2

**DOI:** 10.5256/f1000research.182131.r395641

**Published:** 2025-08-09

**Authors:** Ejemai Eboreime

**Affiliations:** 1 Dalhousie University, Halifax, Nova Scotia, Canada

This conceptual paper tackles an important and underexplored dimension of disaster preparedness in Malaysia: the psychological readiness of individuals facing floods. The authors rightly point out that while the country has developed strong physical preparedness infrastructures, psychological aspects remain neglected—despite the documented mental health consequences of flood-related disasters. In response to this gap, the authors propose a framework grounded in five key psychological predictors: risk perception, outcome expectancy, self-efficacy, anxiety, and social support. Drawing on international literature, they argue for integrating psychological preparedness into Malaysia’s disaster management strategies.

 Overall, the paper addresses a meaningful and timely research gap, and its proposed framework has the potential to inform both future research and policy. The work is conceptually promising and contributes to the growing recognition of mental health within disaster risk reduction. However, the manuscript in its current form does not meet the methodological and scholarly standards required for publication. Major revisions are needed to enhance both the rigor and contextual relevance of the paper.

 The most significant issue lies in the methodology. Although the authors claim to have conducted a “systematic literature review,” the manuscript provides minimal detail on how the review was carried out. Crucial methodological elements are missing, including specific search strategies, defined inclusion and exclusion criteria, article screening and selection processes, and any form of quality appraisal. Without these, the review cannot be independently verified or reproduced, which severely undermines its scientific credibility. To address this, the authors must include detailed database search strings (with dates and applied filters), a PRISMA flow diagram to visualize the selection process, and a clear account of how thematic analysis was conducted. Supplementary materials outlining the full search results and data extraction would also enhance transparency and replicability.

 Additionally, although the framework is presented as tailored to the Malaysian context, it draws predominantly from Western studies and theoretical models. The lack of engagement with Malaysian-specific literature and cultural perspectives weakens the relevance and applicability of the proposed framework. The authors should make greater efforts to incorporate Malaysian government reports, regional studies, and academic work rooted in local contexts. Furthermore, they need to consider cultural dimensions such as collectivist social values and Islamic beliefs that may influence psychological preparedness. A discussion of the potential limitations of applying Western-derived constructs in Malaysia, and how these might be adapted or reinterpreted culturally, is essential to strengthen the manuscript’s contextual grounding.

 In summary, while the conceptual direction of this paper is strong and potentially impactful, it requires substantial revisions to its methodology and contextual analysis before it can be accepted.

Is the work clearly and accurately presented and does it cite the current literature?

Partly

If applicable, is the statistical analysis and its interpretation appropriate?

Not applicable

Are all the source data underlying the results available to ensure full reproducibility?

No

Is the study design appropriate and is the work technically sound?

Partly

Are the conclusions drawn adequately supported by the results?

Partly

Are sufficient details of methods and analysis provided to allow replication by others?

No

Reviewer Expertise:

Disasters and mental health

I confirm that I have read this submission and believe that I have an appropriate level of expertise to state that I do not consider it to be of an acceptable scientific standard, for reasons outlined above.

# Response

**Published:** 2025-10-21

**Authors:** Nur Farhana Lyana Ameruddin

Dear Reviewer,

 We would like to thank you for your thoughtful and constructive feedback on our manuscript titled “Exploring Predictors of Psychological Preparedness for Flood Victims: A Conceptual Framework for Malaysia.” We deeply appreciate your comments and insights, which have guided us in strengthening both the methodological rigor and contextual grounding of this paper.

 Below is our point-by-point response to each comment. All revisions are reflected in the manuscript through track changes, with section and page numbers provided for clarity.

**Feedback 1:** Methodology and Transparency

 The most significant issue lies in the methodology. Although the authors claim to have conducted a “systematic literature review,” the manuscript provides minimal detail on how the review was carried out. Crucial methodological elements are missing, including specific search strategies, defined inclusion and exclusion criteria, article screening and selection processes, and any form of quality appraisal. Without these, the review cannot be independently verified or reproduced, which severely undermines its scientific credibility. To address this, the authors must include detailed database search strings (with dates and applied filters), a PRISMA flow diagram to visualize the selection process, and a clear account of how thematic analysis was conducted. Supplementary materials outlining the full search results and data extraction would also enhance transparency and replicability.

**Response: **Thank you for highlighting the need for greater methodological transparency. We have clarified that the current paper employs a targeted review of disaster preparedness literature, rather than a systematic literature review. The purpose of this approach is to identify key psychological factors and integrate them into a conceptual framework explaining psychological preparedness for floods in Malaysia. Articles were included if they focused on disaster preparedness and examined coping mechanisms, perceptions, or preparedness behaviours related to flood contexts. Studies outside the crisis or disaster scope or those not meeting basic methodological standards were excluded. The conceptual framework was developed by synthesizing findings from the reviewed literature and integrating theories on catastrophic risk reduction and psychological resilience. This approach contributed to a contextually grounded framework describing psychological processes and predictors of preparedness among the Malaysian population. These revisions have been incorporated in the abstract (p. 2) and elaborated in the methods section (p. 16) for clarity.

**Feedback 2:** Contextual Relevance and Malaysian Literature

 Although the framework is presented as tailored to the Malaysian context, it draws predominantly from Western studies and theoretical models. The lack of engagement with Malaysian-specific literature and cultural perspectives weakens the relevance and applicability of the proposed framework. The authors should make greater efforts to incorporate Malaysian government reports, regional studies, and academic work rooted in local contexts. Furthermore, they need to consider cultural dimensions such as collectivist social values and Islamic beliefs that may influence psychological preparedness.

**Response: **We appreciate this valuable comment on enhancing the manuscript’s local and cultural grounding. To address this, we have expanded the discussion to include:

- The geographical vulnerability of Malaysia to floods and the predominance of physical preparedness measures.
- The limited attention to psychological preparedness, emphasizing how collectivist culture and Islamic beliefs may influence coping methods and collective responses to disasters.
- Integration of Malaysian government reports, regional studies, and academic works to support the role of communal and cultural behaviour in disaster contexts.

These additions strengthen the framework’s local relevance and complement international findings on psychological preparedness. Revisions are reflected in p. 3, 4, 5, and 15.

**Feedback 3: **Cultural Adaptation of Western Constructs

 A discussion of the potential limitations of applying Western-derived constructs in Malaysia, and how these might be adapted or reinterpreted culturally, is essential to strengthen the manuscript’s contextual grounding.

**Response: **We have incorporated this recommendation by adding a new section titled “Adaptation of Western Constructs” (p. 18). This section acknowledges that the current framework is primarily based on Western, individualistic perspectives, which may not fully reflect Malaysia’s collectivist cultural context.

 It discusses how psychological constructs such as self-efficacy and social support may be reflected differently in Malaysian society and highlights the need for empirical validation of the framework in future studies. This addition strengthens the paper’s contextual grounding and outlines clear directions for future research.

 We sincerely thank the reviewer for their constructive comments. The revisions made in response have enhanced the methodological clarity, conceptual strength, and cultural relevance of our manuscript.

# Reviewer report: Reviewer response for version 1

**DOI:** 10.5256/f1000research.176090.r367008

**Published:** 2025-03-10

**Authors:** Listyati Setyo Palupi

**Affiliations:** 1 Universitas Airlangga, Surabaya, Indonesia

This article investigates the factors that may predict psychological preparedness among flood victims in Malaysia, a topic of considerable relevance given the increasing frequency and severity of floods, which are recognized as one of the most destructive natural disasters in the country. The authors provide a thorough overview of past flood events in Malaysia and their associated impacts. However, while studies on psychological preparedness for flooding in the Malaysian context have been limited, the authors could strengthen their analysis by synthesizing findings from prior research on psychological preparedness for disasters more generally. Notably, studies such as Boylan's (2016) investigation of psychological preparedness in the context of wildfires, and Zulch's (2019) broader examination of psychological preparedness for natural hazards, could offer valuable insights to enhance the current study.

 The article employs a systematic literature review methodology, yet the authors do not provide sufficient detail regarding the process used to identify, evaluate, and synthesize relevant research on psychological preparedness for flood victims. To improve the transparency and rigor of the review process, the authors could adopt established guidelines, such as the PRISMA (Preferred Reporting Items for Systematic Reviews and Meta-Analyses) framework, which is widely regarded as the gold standard for conducting systematic reviews. Adhering to PRISMA standards would enhance the reproducibility and reliability of the review process and ensure alignment with recognized methodological best practices.

 Although the study would benefit from improvements in methodological transparency, particularly with respect to the reliability and rigor of the systematic review process, it nonetheless holds significant potential to contribute to disaster risk reduction efforts, particularly in the domain of psychological preparedness for natural disasters. By addressing these methodological gaps, the study could provide valuable insights that inform the development of more effective and comprehensive disaster preparedness strategies.

Is the work clearly and accurately presented and does it cite the current literature?

Partly

If applicable, is the statistical analysis and its interpretation appropriate?

Not applicable

Are all the source data underlying the results available to ensure full reproducibility?

No source data required

Is the study design appropriate and is the work technically sound?

Partly

Are the conclusions drawn adequately supported by the results?

Partly

Are sufficient details of methods and analysis provided to allow replication by others?

No

Reviewer Expertise:

Psychological Preparedness for Disaster, Disaster Preparedness, Disaster Management, Environmental Psychology, Applied Psychology, Social Psychology.

I confirm that I have read this submission and believe that I have an appropriate level of expertise to confirm that it is of an acceptable scientific standard, however I have significant reservations, as outlined above.

# Response

**Published:** 2025-05-15

**Authors:** Nur Farhana Lyana Ameruddin

Thank you for your suggestions.

 In response, we have incorporated additional studies that explore psychological preparedness in the context of various natural hazards, including Boylan (2016) on page 7, as well as Clode (2010), Boylan and Lawrence (2020), and Zulch (2019) on page 4. These studies provide broader theoretical perspectives and empirical findings that enrich the foundation of our conceptual framework.

 We would also like to clarify that this paper adopts a conceptual approach, aimed at developing a theoretical framework rather than conducting a systematic literature review. While we recognize the value of systematic review protocols such as PRISMA in enhancing methodological rigor and transparency, these are typically applied to systematic reviews rather than conceptual papers. Nonetheless, to strengthen the clarity and transparency of our literature exploration process, we have included a more detailed description of how the literature was selected and synthesized in the revised methodology section on page 16.

# Reviewer report: Reviewer response for version 1

**DOI:** 10.5256/f1000research.176090.r367014

**Published:** 2025-03-06

**Authors:** Aloysius Gunadi Brata

**Affiliations:** 1 Atma Jaya Yogyakarta University, Depok, Indonesia

This paper examines a critical issue concerning flood victims, specifically within Malaysia.

 It investigates the following research questions: 1) What are the key predictors of psychological preparedness for floods among the Malaysian population? and 2) How do individual and community-level factors affect psychological preparedness for floods?

 To enhance readability and comprehension, the discussion should be restructured to clearly address the "what" and "how" elements of these questions. Ideally, the conclusion will offer a succinct overview of the primary findings related to both research questions.

Is the work clearly and accurately presented and does it cite the current literature?

Yes

If applicable, is the statistical analysis and its interpretation appropriate?

Not applicable

Are all the source data underlying the results available to ensure full reproducibility?

Yes

Is the study design appropriate and is the work technically sound?

Yes

Are the conclusions drawn adequately supported by the results?

Yes

Are sufficient details of methods and analysis provided to allow replication by others?

Yes

Reviewer Expertise:

social impacts of natural disasters

I confirm that I have read this submission and believe that I have an appropriate level of expertise to confirm that it is of an acceptable scientific standard, however I have significant reservations, as outlined above.

# Response

**Published:** 2025-05-15

**Authors:** Nur Farhana Lyana Ameruddin

Thank you for the valuable feedback. In response to your suggestion, the discussion section has been restructured to improve clarity and alignment with the two research questions. Specifically, new subheadings have been introduced: *“Key Predictors of Psychological Preparedness towards Floods”* (page 9) and *“Individual and Community-Level Factors in Psychological Preparedness for Floods”* (page 14). These changes are intended to enhance readability and allow for a clearer discussion of the “what” and “how” elements of the research questions.
